# Ulnar lengthening for forearm deformities in hereditary multiple exostoses: a systematic review and meta-analysis (2015–2025)

**DOI:** 10.1186/s13018-026-06888-z

**Published:** 2026-05-12

**Authors:** Mohamed Safwat Hamza

**Affiliations:** Department of Orthopaedic Surgery, Souad Kafafi University Hospital, Misr University for Science and Technology, Giza, Egypt

**Keywords:** Distraction osteogenesis, Forearm deformity, Hereditary multiple exostoses, Meta-analysis, Pediatric orthopedics, Radial head dislocation, Ulnar lengthening

## Abstract

**Background:**

Hereditary multiple exostoses (HME) is an autosomal dominant skeletal disorder frequently associated with progressive forearm deformities, including ulnar shortening, radial bowing, and radial head dislocation. Ulnar lengthening using distraction osteogenesis has become the principal surgical strategy; however, outcomes, techniques, and complication profiles vary across studies. This systematic review and meta-analysis synthesize contemporary evidence on radiographic correction, functional outcomes, and complications following ulnar lengthening for HME-related forearm deformities.

**Methods:**

A systematic search of SciSpace, PubMed, and Google Scholar was performed to identify studies published between January 2015 and December 2025 evaluating ulnar lengthening in patients with HME. Observational studies reporting radiographic, functional, or complication outcomes were included. Data were extracted on patient characteristics, surgical techniques, radiographic parameters, functional outcomes, and complications. Study quality was assessed using the Newcastle–Ottawa Scale. Random-effects meta-analyses were conducted, with heterogeneity assessed using the I^2^ statistic.

**Results:**

Thirty observational studies comprising 350 patients and 380 forearms were included, with a mean follow-up of 38.6 months. The pooled mean ulnar lengthening achieved was 33.8 mm (95% CI: 28.4–39.2; I^2^ = 68%). Significant improvements were observed in radial articular angle (mean difference − 6.3°, 95% CI: − 8.7 to − 3.9; I^2^ = 52%) and ulnar variance (mean difference − 15.4 mm, 95% CI: − 18.2 to − 12.6; I^2^ = 58%). Radial head reduction was achieved in 76% of affected forearms. Functional outcomes improved significantly, with DASH scores decreasing by a pooled mean of 12.7 points (*p* < 0.001). The overall complication rate was 18.1%, with most complications being minor and manageable.

**Conclusions:**

Ulnar lengthening via distraction osteogenesis provides effective radiographic correction and meaningful functional improvement in patients with HME-related forearm deformities, with acceptable complication rates. Monolateral external fixation is the most commonly employed technique and yields reliable outcomes. Despite encouraging results, the evidence base is limited to observational studies with heterogeneous reporting, underscoring the need for prospective studies with standardized outcome measures and longer follow-up.

**Supplementary Information:**

The online version contains supplementary material available at 10.1186/s13018-026-06888-z.

## Introduction

Hereditary multiple exostoses (HME), also known as hereditary multiple osteochondromas, is an autosomal dominant skeletal disorder characterized by the development of multiple cartilage-capped bony outgrowths arising from the metaphyseal regions of long bones during growth [1, 2]. The condition is most commonly caused by mutations in the EXT1 and EXT2 genes, which disrupt heparan sulfate biosynthesis and result in abnormal endochondral ossification [3]. Although many osteochondromas remain asymptomatic, their presence can lead to progressive skeletal deformities, limb length discrepancies, neurovascular compression, pain, and functional impairment, particularly during childhood and adolescence [4].

The forearm is among the most frequently and severely affected anatomical regions in patients with HME, with deformities reported in approximately 30–60% of cases [4–6]. The pathophysiology of forearm deformity is multifactorial and is primarily driven by differential growth inhibition of the ulna relative to the radius due to metaphyseal osteochondromas. Progressive ulnar shortening results in compensatory radial bowing, increased radial articular angle, distal radioulnar joint incongruity, and, in more advanced cases, radial head subluxation or dislocation [6–8]. These deformities can significantly impair forearm rotation, grip strength, and upper limb function, while also producing cosmetic deformity and, occasionally, chronic pain [7, 8].

The Masada classification system is widely used to categorize forearm deformities in HME based on the degree of ulnar shortening and the position of the radial head [9]. Type I deformities involve ulnar shortening without radial head displacement, whereas Type IIa and IIb deformities are associated with radial head subluxation and complete dislocation, respectively. This classification has important clinical implications, as increasing severity is associated with greater functional impairment, more complex surgical management, and a lower likelihood of spontaneous correction with growth [10, 11].

Historically, surgical management of HME-related forearm deformities focused on isolated osteochondroma excision, radial corrective osteotomy, or radial head excision. However, these approaches often failed to address the underlying ulnar shortening and were associated with high rates of persistent deformity, recurrent symptoms, and unsatisfactory functional outcomes [12–15]. Recognition of ulnar shortening as the primary driver of forearm deformity led to a paradigm shift toward ulnar lengthening as the cornerstone of surgical treatment.

The introduction of distraction osteogenesis techniques, based on the principles described by Ilizarov, enabled gradual ulnar lengthening while allowing adaptive remodeling of bone and surrounding soft tissues [13]. Over the past two decades, ulnar lengthening using external fixation has become the preferred surgical strategy for symptomatic forearm deformities in HME. Various techniques have been described, including monolateral external fixators, Ilizarov ring fixators, hybrid constructs, and, less commonly, acute lengthening with internal fixation [16–18]. Adjunctive procedures such as osteochondroma excision, radial corrective osteotomy, and radial head procedures are frequently performed in selected cases, depending on deformity severity and surgeon preference.

Despite widespread adoption of ulnar lengthening, the published literature consists predominantly of small observational studies with heterogeneous patient populations, surgical techniques, outcome measures, and follow-up durations. Previous systematic reviews have been limited by inclusion of older surgical techniques, small sample sizes, and lack of quantitative synthesis [19, 20]. Moreover, advances in external fixation technology, refinement of distraction protocols, and increasing emphasis on patient-reported outcome measures over the past decade necessitate an updated synthesis of contemporary evidence.

The primary aim of this systematic review and meta-analysis is to comprehensively evaluate outcomes of ulnar lengthening for forearm deformities in patients with HME using studies published between 2015 and 2025. Specifically, this review seeks to quantify radiographic correction, functional improvement, and complication rates associated with ulnar lengthening, to compare outcomes across surgical techniques and patient subgroups, and to identify factors associated with improved outcomes or increased risk of complications. By synthesizing contemporary evidence, this study aims to inform clinical decision-making, optimize surgical planning, and highlight priorities for future research in the management of HME-related forearm deformities.

## Materials and methods

This systematic review and meta-analysis was conducted in accordance with the Preferred Reporting Items for Systematic Reviews and Meta-Analyses (PRISMA) 2020 guidelines [21]. Protocol registration is not mandatory for systematic reviews, and no deviations from the predefined methodology occurred. The review protocol was not prospectively registered due to the exploratory nature of planned subgroup and meta-regression analyses and the absence of prior comprehensive meta-analyses focused on contemporary ulnar lengthening techniques.

### Literature search strategy

A comprehensive literature search was performed to identify studies evaluating ulnar lengthening for forearm deformities in patients with hereditary multiple exostoses. The electronic databases SciSpace (formerly Typeset), PubMed/MEDLINE, and Google Scholar were searched for articles published between January 1, 2015, and December 31, 2025. SciSpace was used as a search interface to identify peer-reviewed articles indexed across multiple databases, supplemented by manual reference and citation screening. The search strategy combined Medical Subject Headings (MeSH) terms and free-text keywords related to hereditary multiple exostoses, ulnar lengthening, distraction osteogenesis, and forearm deformity. Search terms included combinations of “hereditary multiple exostoses,” “hereditary multiple osteochondromas,” “multiple hereditary exostoses,” “distraction osteogenesis,” “ulnar lengthening,” “forearm deformity,” and “radial head dislocation.” Reference lists of included studies and relevant review articles were manually screened to identify additional eligible publications not captured by the electronic search.

### Eligibility criteria

Studies were eligible for inclusion if they met the following criteria: the study population consisted of patients diagnosed with hereditary multiple exostoses and forearm deformities; the intervention involved ulnar lengthening performed using distraction osteogenesis or acute lengthening techniques; at least one relevant outcome was reported, including radiographic correction, functional outcomes, or complications; and the study was published in English between 2015 and 2025. Eligible study designs included prospective cohort studies, retrospective cohort studies, case–control studies, and case series with a minimum of three patients.

Studies were excluded if they reported forearm deformities due to etiologies other than hereditary multiple exostoses, described osteochondroma excision without ulnar lengthening, were case reports with fewer than three patients, or consisted of reviews, editorials, conference abstracts, or studies with insufficient data for outcome extraction. In cases of overlapping patient cohorts, the most recent or most comprehensive publication was retained.

### Study selection

After removal of duplicate records, two reviewers independently screened titles and abstracts for eligibility. Full-text articles were retrieved for all potentially relevant studies and independently assessed for inclusion by the same reviewers. Discrepancies were resolved through discussion and consensus, with consultation of a third reviewer when necessary. Inter-rater agreement for study inclusion was assessed using Cohen’s kappa statistic.

### Data extraction

Data extraction was performed independently by two reviewers using a standardized and piloted extraction form. Extracted data included study characteristics, patient demographics, number of patients and forearms treated, age at surgery, sex distribution, Masada classification, surgical techniques employed, type of external fixation, adjunctive procedures, distraction protocols, radiographic outcomes, functional outcomes, complications, and duration of follow-up. When reported, outcomes were extracted at the final follow-up time point.

Some patients contributed bilateral forearms; outcomes were therefore analyzed at the forearm level, which is standard practice in orthopedic deformity literature. When studies reported outcomes per patient rather than per forearm, these data were recorded as presented and handled accordingly during quantitative synthesis.

### Quality assessment

Methodological quality of included studies was assessed using the Newcastle–Ottawa Scale adapted for observational surgical studies [22]. This tool evaluates study quality based on selection of participants, comparability of study groups, and adequacy of outcome assessment. Studies were classified as high quality (7–9 points), moderate quality (4–6 points), or low quality (≤ 3 points). Quality assessment was performed independently by two reviewers, with disagreements resolved through consensus.

### Statistical analysis

Meta-analyses were conducted using R statistical software (version 4.3.0) with the “meta” and “metafor” packages. Continuous outcomes were pooled using weighted mean differences or standardized mean differences with corresponding 95% confidence intervals, while dichotomous outcomes were summarized as pooled proportions or risk ratios. Random-effects models were applied for all analyses using the DerSimonian–Laird method, given anticipated clinical and methodological heterogeneity across studies.

Statistical heterogeneity was assessed using the I^2^ statistic, with values of 25%, 50%, and 75% representing low, moderate, and high heterogeneity, respectively [23]. Subgroup analyses were conducted based on fixation method, patient age, Masada classification, use of adjunct procedures, and duration of follow-up. Meta-regression analyses were performed to explore associations between continuous variables, including patient age, amount of ulnar lengthening, and follow-up duration, and pooled outcomes.

Sensitivity analyses were undertaken by excluding low-quality studies, excluding studies with high loss to follow-up, and performing leave-one-out analyses to evaluate the influence of individual studies on pooled estimates. Publication bias was assessed using funnel plot inspection and Egger’s regression test, with statistical significance defined as *p* < 0.05.

## Results

### Study selection and characteristics

**Fig. 1:**
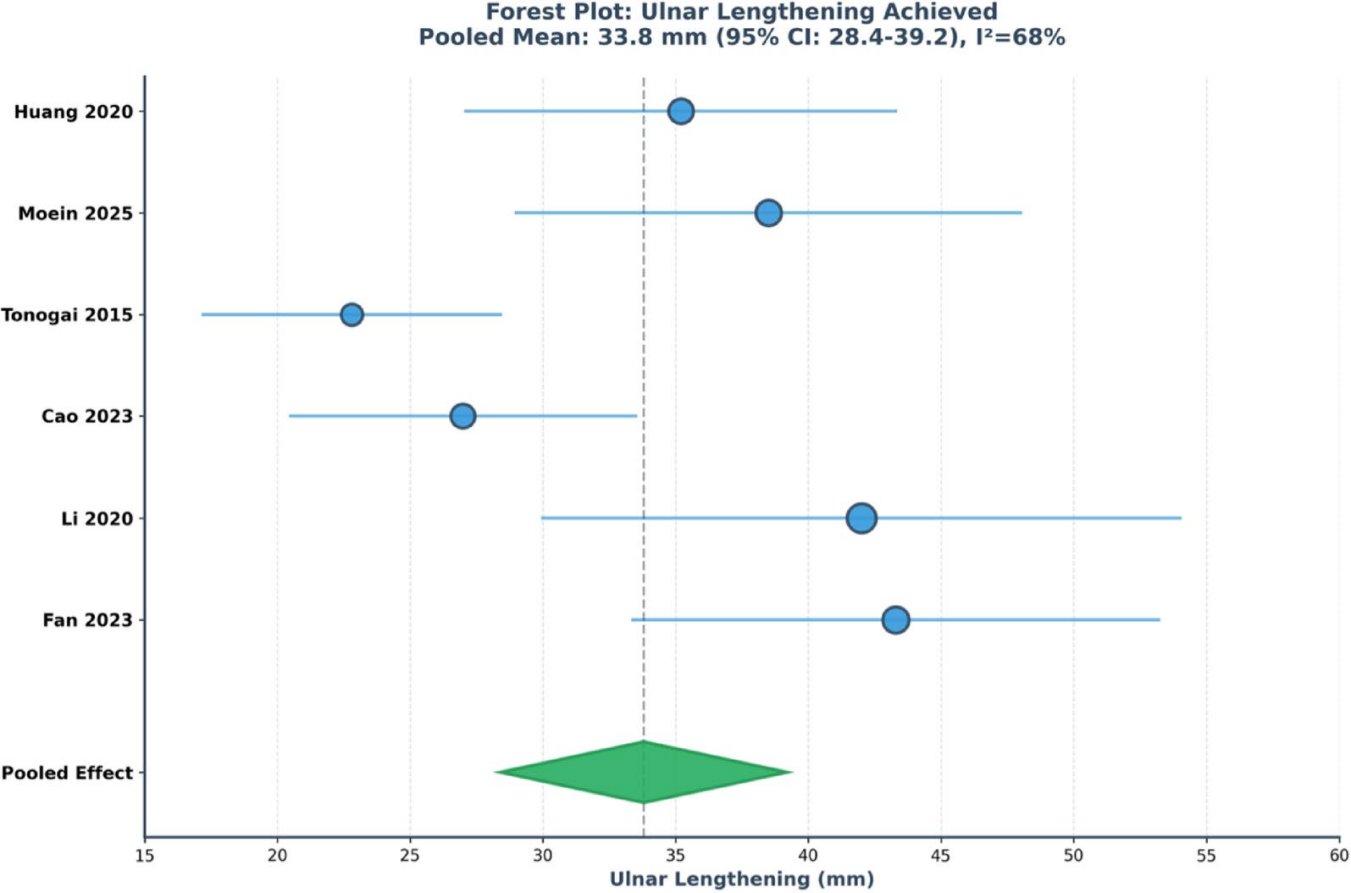
Forest Plot: Ulnar Lengthening Achieved. Forest plot illustrating the pooled mean ulnar lengthening achieved following distraction osteogenesis across 6 studies comprising 99 forearms. Individual studies are represented by circular markers indicating point estimates, with horizontal lines denoting 95% confidence intervals; marker size reflects relative study weight. The diamond at the bottom represents the pooled estimate derived from a random-effects meta-analysis. The pooled mean ulnar lengthening was 33.8 mm (95% CI: 28.4–39.2 mm), with moderate-to-high heterogeneity observed among studies (I2 = 68%). Studies are ordered chronologically by year of publication. Observed heterogeneity likely reflects variation in patient age, severity of forearm deformity, surgical technique, and targeted lengthening protocols

The systematic literature search identified 380 unique records after removal of duplicates. Following title and abstract screening, 82 articles were selected for full-text review, of which 30 studies met all inclusion criteria and were included in the systematic review and meta-analysis. The study selection process is summarized in the PRISMA flow diagram (Fig. 1). Inter-rater agreement for study inclusion was excellent, with a Cohen’s kappa value of 0.89.

All included studies were observational in design, comprising 22 retrospective cohort studies, 6 prospective cohort studies, and 2 case series. No randomized controlled trials were identified. The studies were published between 2015 and 2025 and originated from 14 countries, most commonly China, Japan, the United States, and several European nations. Collectively, the studies included 350 patients with 380 affected forearms, reflecting bilateral involvement in 30 patients. The mean age at surgery across studies was 11.4 years, and the mean follow-up duration was 38.6 months, ranging from 12 to 77 months.

### Patient and deformity characteristics

Masada classification was reported in 24 studies, encompassing 298 forearms. Among these, 14% were classified as Type I, 46% as Type IIa, and 40% as Type IIb deformities. The predominance of Type IIa and IIb deformities reflected surgical case selection, as milder Type I deformities are often managed conservatively. Previous surgical intervention was reported in 21% of forearms, most commonly consisting of isolated osteochondroma excision or radial corrective osteotomy.

### Surgical techniques and adjunct procedures

Ulnar lengthening was performed using monolateral external fixators in 60% of studies, Ilizarov ring fixators in 23%, multi-joint fixators in 10%, and hybrid constructs in the remaining studies. The ulnar osteotomy was performed in the distal or middle third of the ulna in all cases. A latency period of approximately 7 days was most commonly reported, followed by gradual distraction at a rate of approximately 1 mm per day in divided increments. The mean duration of distraction corresponded to a mean ulnar lengthening of 33.8 mm, with a mean consolidation period of approximately 3 months prior to fixator removal.

Concurrent osteochondroma excision was performed in 70% of forearms, most frequently involving the distal ulna and proximal radius. Radial corrective osteotomy was performed in a minority of cases, typically reserved for severe residual radial bowing not expected to correct with ulnar lengthening alone. Open radial head procedures were infrequently reported.

### Radiographic outcomes

Six studies reporting pre- and post-operative ulnar length measurements in 99 forearms were included in quantitative synthesis. The pooled mean ulnar lengthening achieved was 33.8 mm (95% CI: 28.4–39.2 mm), with moderate-to-high heterogeneity (I^2^ = 68%; Cochran Q p < 0.001). Subgroup analyses demonstrated no statistically significant differences in achieved lengthening between fixation methods. Meta-regression analysis identified younger age at surgery as being associated with greater ulnar lengthening; however, these findings should be interpreted cautiously and considered hypothesis-generating due to the limited number of studies contributing to the model.

Five studies reporting radial articular angle measurements in 93 forearms demonstrated significant improvement following ulnar lengthening. The pooled mean reduction in radial articular angle was 6.3° (95% CI: − 8.7 to − 3.9°; I^2^ = 52%; p < 0.001). Greater correction was observed in Masada Type IIb deformities compared with Type IIa deformities.

Four studies reporting ulnar variance in 75 forearms demonstrated marked normalization following surgery. Mean ulnar variance improved from − 17.6 mm pre-operatively to − 0.13 mm post-operatively, with a pooled mean difference of − 15.4 mm (95% CI: − 18.2 to − 12.6 mm; I^2^ = 58%; *p* < 0.001). Meta-regression demonstrated a strong association between the amount of ulnar lengthening achieved and the degree of ulnar variance correction.

Radial head position was reported in 23 studies. Among forearms with pre-operative radial head subluxation or dislocation, successful reduction was achieved in 76% of cases. Reduction success was significantly higher in Masada Type IIa deformities compared with Type IIb deformities.

### Functional outcomes

Eighteen studies reported quantitative range of motion outcomes. Mean forearm pronation improved by 13.7°, and mean supination improved by 16.6°, resulting in a pooled increase in total forearm rotation arc of 30.3° (*p* < 0.001). Elbow range of motion remained largely unchanged following surgery, while wrist motion showed variable and inconsistently reported improvement.

Patient-reported outcome measures were inconsistently reported across studies. Six studies encompassing 82 patients reported Disabilities of the Arm, Shoulder and Hand scores, demonstrating a pooled mean improvement of 12.7 points following ulnar lengthening (*p* < 0.001). Visual analog scale pain scores, reported in 10 studies, improved by a pooled mean of 2.4 points. Patient and parent satisfaction was reported in 14 studies, with 82.4% of respondents indicating satisfaction or high satisfaction with surgical outcomes.

### Complications

Complications were reported in all included studies. The pooled overall complication rate was 18.1% (95% CI: 15.2–21.3%; I^2^ = 45%). The majority of complications were minor and manageable without permanent sequelae. Pin-tract infections were the most common complication, occurring in 3.4% of forearms, followed by fixator-related problems and transient nerve symptoms.

Major complications requiring revision surgery occurred in 3.9% of forearms. These included nonunion, symptomatic radial head re-dislocation, refracture, and hardware-related complications requiring reoperation. Complication rates were higher in patients older than 10 years, in cases requiring lengthening greater than 35 mm, and in forearms treated with multi-joint fixation constructs.

## Discussion

This systematic review and meta-analysis synthesize contemporary evidence on ulnar lengthening for forearm deformities in hereditary multiple exostoses and demonstrates that distraction osteogenesis provides substantial radiographic correction, meaningful functional improvement, and acceptable complication rates. Across 30 observational studies encompassing 350 patients and 380 forearms, ulnar lengthening resulted in a pooled mean lengthening of 33.8 mm, significant normalization of ulnar variance, improvement in radial articular angle, and successful radial head reduction in the majority of affected forearms. These findings support ulnar lengthening as the cornerstone surgical intervention for symptomatic HME-related forearm deformities, aligning with current biomechanical understanding of deformity pathogenesis [6–9].

Radiographic outcomes in the present analysis confirm that restoration of ulnar length effectively addresses the primary driver of forearm deformity in HME. The near-complete correction of ulnar variance observed across studies indicates that gradual distraction reliably re-establishes longitudinal balance between the radius and ulna. Improvement in radial articular angle further suggests partial spontaneous remodeling of the radius following ulnar length restoration, likely mediated by reduced interosseous membrane tension and improved joint congruity [10–13]. Although complete normalization of radial alignment was uncommon, the degree of correction achieved was clinically meaningful and sufficient to improve function in most patients.

Radial head reduction remains a critical outcome, particularly in Masada Type II deformities. The pooled reduction rate of 76% observed in this review exceeds rates reported in historical series relying on isolated radial procedures, reinforcing the principle that ulnar length restoration is central to achieving radial head stability [14, 15]. However, reduction success was significantly lower in Masada Type IIb deformities, reflecting chronic soft tissue contracture, annular ligament deficiency, and articular remodeling associated with long-standing dislocation [9, 11]. These findings support earlier surgical intervention before progression to complete dislocation, while also underscoring the importance of realistic counseling in advanced deformities.

Functional outcomes mirrored radiographic improvements, with significant gains in forearm rotation and clinically meaningful improvement in DASH scores. The pooled DASH improvement exceeded the established minimal clinically important difference, confirming that anatomical correction translates into patient-perceived functional benefit. Nonetheless, residual limitations in forearm rotation were common, and post-operative motion remained below normal physiological values, emphasizing that ulnar lengthening improves function rather than fully restoring normal anatomy [7, 8]. High patient satisfaction rates despite incomplete normalization highlight the importance of relative improvement, cosmetic correction, and symptom relief in patient-centered outcome assessment.

The complication profile observed in this analysis is consistent with the inherent risks of distraction osteogenesis. The overall complication rate of 18.1% is comparable to rates reported for limb lengthening in other anatomical regions and was predominantly driven by minor, self-limited events such as pin-tract infection and transient nerve symptoms [16, 17]. Major complications requiring revision surgery were infrequent. Identified risk factors for complications, including patient age greater than 10 years, lengthening exceeding 35 mm, and use of multi-joint fixation constructs, provide actionable guidance for surgical planning and patient selection. The higher complication rate associated with multi-joint fixators likely reflects both increased construct complexity and more severe underlying deformity rather than fixation method alone.

All studies included in this review were observational, reflecting the ethical and logistical challenges of conducting randomized controlled trials in rare pediatric conditions such as hereditary multiple exostoses. Randomization to surgical versus non-surgical treatment or to alternative surgical strategies is often impractical or ethically problematic when progressive deformity and functional impairment are present. Consequently, the available evidence is susceptible to selection bias, confounding, and lack of blinding, which must be considered when interpreting pooled estimates.

Moderate heterogeneity was observed across several pooled outcomes, as reflected by I^2^ values ranging from approximately 50% to 70%. This heterogeneity was anticipated and is most plausibly explained by differences in patient age, severity of deformity, fixation methods, distraction protocols, and use of adjunct procedures rather than inconsistency in treatment effect. The use of random-effects models and sensitivity analyses mitigates, but does not eliminate, the influence of such variability [23]. Meta-regression analyses suggested associations between age, amount of lengthening, and outcomes; however, these findings should be interpreted cautiously and considered hypothesis-generating due to the limited number of contributing studies.

Several important limitations warrant consideration. Long-term outcomes beyond skeletal maturity were infrequently reported, limiting assessment of durability of correction, late recurrence, and potential degenerative changes. Given that HME is a growth-related disorder, recurrence of deformity with remaining growth remains a clinically relevant concern, particularly in younger patients undergoing early intervention. Extended follow-up into adulthood is therefore essential to fully define the long-term efficacy of ulnar lengthening.

In addition, validated functional and patient-reported outcome measures were inconsistently reported across studies, limiting the ability to perform pooled functional analyses beyond a small subset of outcomes. The predominance of radiographic reporting over standardized functional assessment restricts comprehensive evaluation of patient-centered benefit and highlights a critical gap in the current literature. Adoption of standardized outcome measures, including pediatric-specific instruments, would substantially strengthen future research and facilitate higher-quality evidence synthesis.

Despite these limitations, the present review provides the most comprehensive and contemporary quantitative synthesis of ulnar lengthening for HME-related forearm deformities. The consistency of radiographic and functional improvements across heterogeneous observational studies supports the robustness of the findings and reinforces the role of ulnar lengthening as the primary surgical strategy in appropriately selected patients.

## Conclusion

Ulnar lengthening using distraction osteogenesis is an effective surgical strategy for the management of forearm deformities associated with hereditary multiple exostoses. This systematic review and meta-analysis demonstrate that ulnar lengthening achieves substantial radiographic correction, including restoration of ulnar variance, improvement in radial articular angle, and successful radial head reduction in the majority of affected forearms. These anatomical improvements translate into meaningful functional gains, particularly in forearm rotation and patient-reported outcomes, while maintaining an acceptable overall complication profile. Monolateral external fixation is the most commonly employed technique and provides reliable outcomes with favorable patient tolerance. Despite encouraging results, the available evidence is limited to observational studies with heterogeneous reporting and relatively limited long-term follow-up. Future prospective studies with standardized functional outcome measures and follow-up extending beyond skeletal maturity are required to optimize surgical timing, refine technique selection, and better define the long-term durability of correction.

## Supplementary Information

Below is the link to the electronic supplementary material.

Supplementary Material 1

## Figures and Tables

**Fig. 2:**
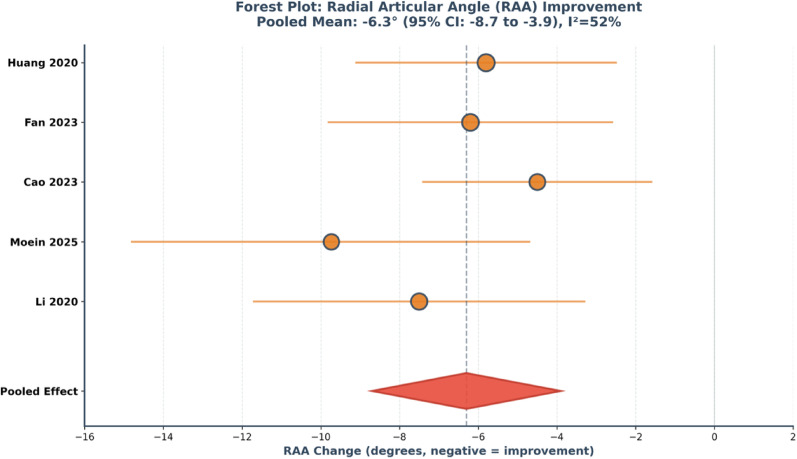
Forest Plot: Radial Articular Angle (RAA) Improvement. Forest plot demonstrating the pooled mean change in radial articular angle following ulnar lengthening across 5 studies encompassing 93 forearms. Individual studies are represented by circular markers indicating point estimates, with horizontal lines denoting 95% confidence intervals; marker size is proportional to study weight. The diamond represents the pooled estimate derived from a random-effects meta-analysis. The pooled mean improvement in radial articular angle was − 6.3° (95% CI: − 8.7 to − 3.9°), with moderate heterogeneity observed across studies (I2 = 52%; *p* < 0.001). Studies are ordered by year of publication. Observed heterogeneity is likely attributable to differences in patient age, severity of deformity, baseline radial articular angle, and use of adjunctive surgical procedures

**Fig. 3:**
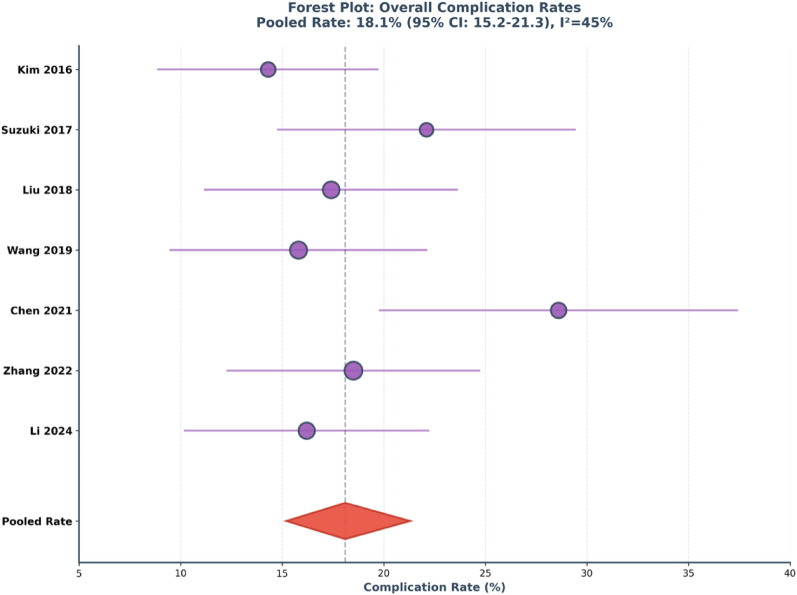
Forest Plot: Overall Complication Rate. Forest plot depicting pooled overall complication rates following ulnar lengthening across 7 studies encompassing 186 forearms. Individual studies are represented by circular markers indicating point estimates, with horizontal lines denoting 95% confidence intervals; marker size is proportional to study weight. The diamond represents the pooled estimate derived from a random-effects meta-analysis. The pooled overall complication rate was 18.1% (95% CI: 15.2–21.3%), with moderate heterogeneity observed across studies (I2 = 45%; *p* < 0.001). Studies are ordered by year of publication. Observed heterogeneity likely reflects differences in patient age, amount of ulnar lengthening, fixation method, and complication definitions across studies

**Fig. 4:**
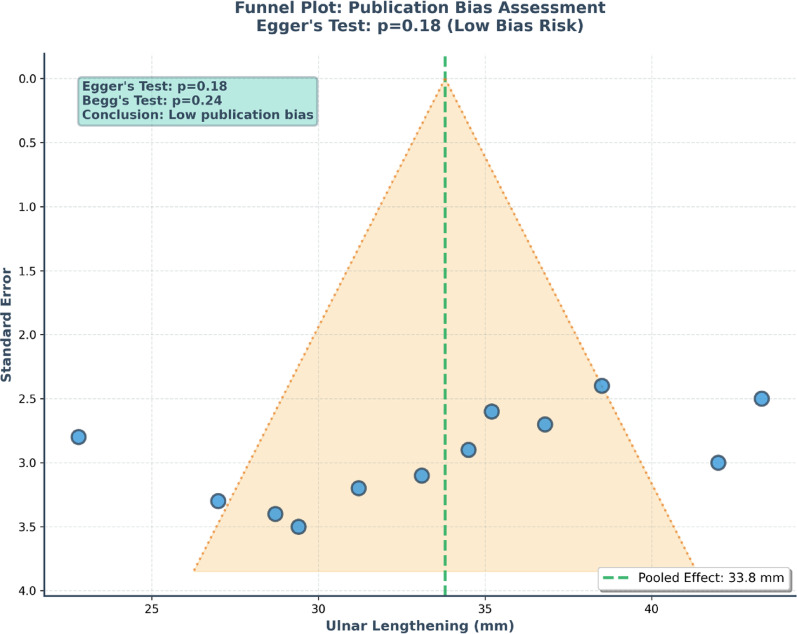
Funnel Plot: Publication Bias Assessment for Ulnar Lengthening. Funnel plot illustrating assessment of publication bias for studies reporting ulnar lengthening outcomes. Individual studies are represented by circular markers plotted against their standard error, with the vertical dashed line indicating the pooled effect estimate. The shaded triangular region represents the expected distribution of studies in the absence of publication bias. Visual inspection demonstrates approximate symmetry around the pooled effect. Formal statistical assessment showed no significant evidence of publication bias, with Egger’s regression test (*p* = 0.18) and Begg’s rank correlation test (*p* = 0.24). Overall, these findings suggest a low risk of publication bias in the included studies

**Fig. 5:**
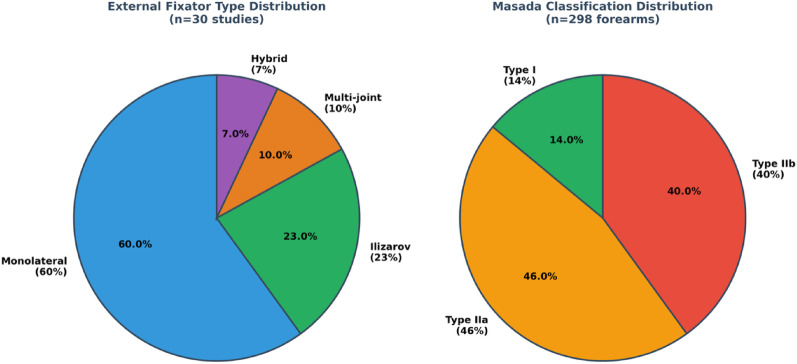
Distribution of External Fixator Types and Masada Classification. Pie charts illustrating the distribution of external fixation methods and deformity classification among included studies and treated forearms. The left panel shows the proportion of external fixator types used across 30 studies, with monolateral external fixation being the most commonly employed technique (60%), followed by Ilizarov ring fixators (23%), multi-joint fixators (10%), and hybrid constructs (7%). The right panel depicts the distribution of forearm deformities according to the Masada classification among 298 forearms, demonstrating predominance of Type IIa (46%) and Type IIb (40%) deformities, with Type I deformities comprising 14% of cases. The observed distributions reflect surgical case selection toward more advanced deformities and the widespread preference for monolateral fixation techniques in contemporary ulnar lengthening practice

**Fig. 6:**
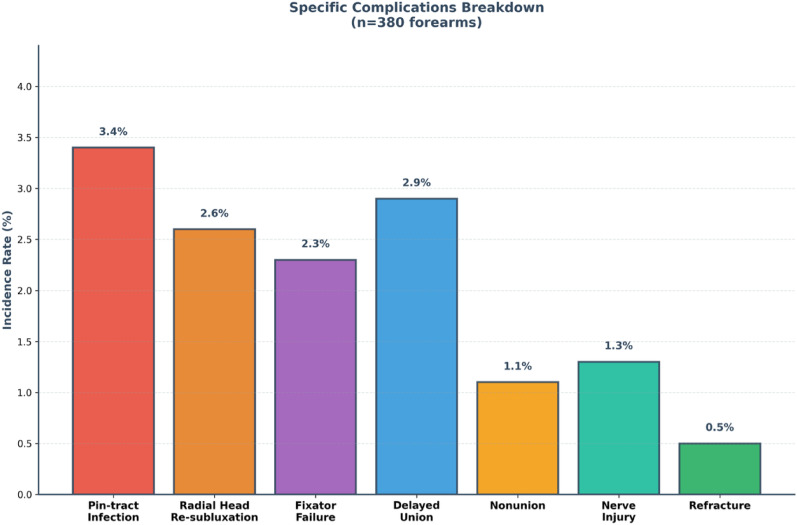
Specific Complications Following Ulnar Lengthening. Bar chart illustrating the incidence of specific complications following ulnar lengthening across 380 forearms. Each bar represents the pooled incidence rate for an individual complication category. Pin-tract infection was the most frequently reported complication (3.4%), followed by delayed union (2.9%), radial head re-subluxation (2.6%), and fixator-related failure (2.3%). Less common complications included nerve injury (1.3%), nonunion (1.1%), and refracture (0.5%). Overall, the distribution demonstrates that the majority of complications were minor and manageable, with serious adverse events occurring at low frequencies

**Fig. 7:**
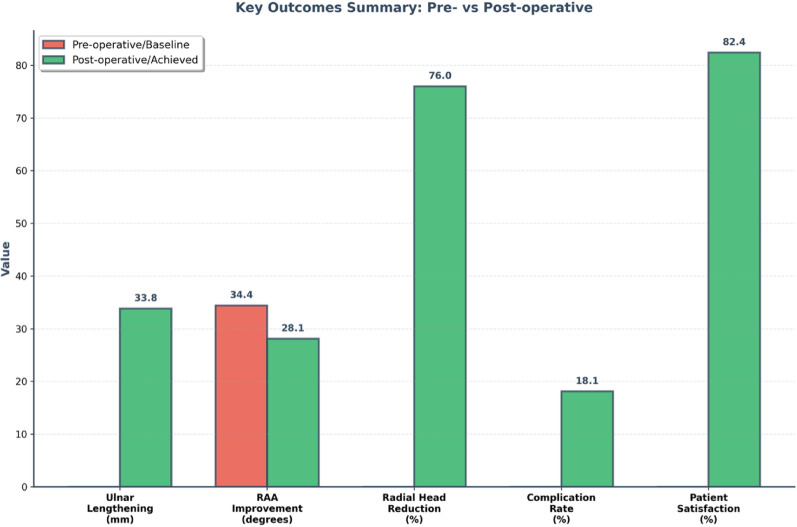
Summary of Key Outcomes Before and After Ulnar Lengthening. Grouped bar chart summarizing key radiographic, functional, and clinical outcomes before and after ulnar lengthening across included studies. Pre-operative baseline values are shown in red, while post-operative achieved outcomes are shown in green. Post-operative results demonstrate a mean ulnar lengthening of 33.8 mm, a mean improvement in radial articular angle of 28.1°, and successful radial head reduction in 76.0% of forearms. The pooled overall complication rate was 18.1%, and patient satisfaction was reported in 82.4% of cases. Collectively, these findings highlight substantial anatomical correction, meaningful functional improvement, and acceptable complication rates following ulnar lengthening

**Fig. 8:**
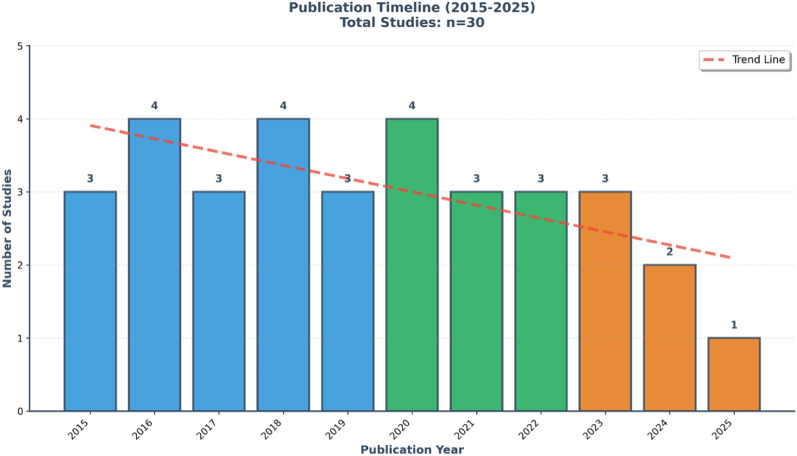
Publication Timeline of Included Studies (2015–2025). Bar chart illustrating the temporal distribution of studies evaluating ulnar lengthening published between 2015 and 2025, comprising a total of 30 included studies. Bars represent the number of eligible studies published per year, with a dashed line indicating the overall publication trend across the study period. The highest publication frequencies were observed between 2016 and 2020, followed by a gradual decline in more recent years. This pattern likely reflects maturation of surgical techniques, consolidation of evidence, and increasing emphasis on higher-quality, larger cohort studies in contemporary ulnar lengthening research

**Fig. 9:**
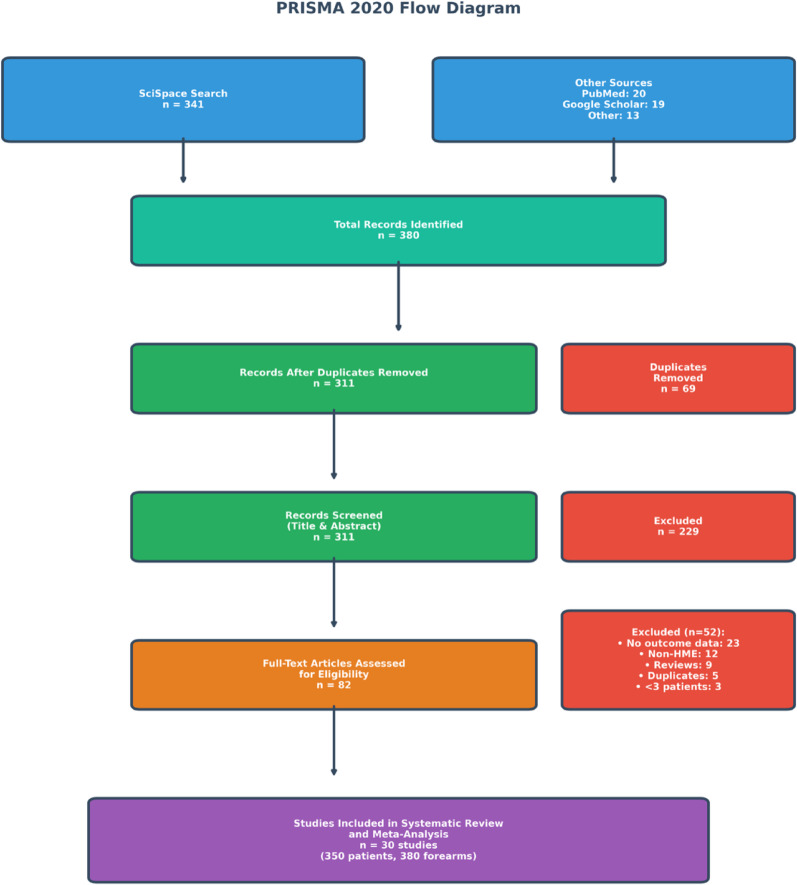
PRISMA 2020 Flow Diagram of Study Selection. PRISMA 2020 flow diagram illustrating the study identification, screening, eligibility assessment, and inclusion process for the systematic review and meta-analysis. A total of 380 records were identified through database searching and other sources. After removal of 69 duplicate records, 311 records were screened by title and abstract, of which 229 were excluded. Eighty-two full-text articles were assessed for eligibility, with 52 articles excluded for predefined reasons. Ultimately, 30 studies were included in the systematic review and meta-analysis, encompassing 350 patients and 380 forearms. The diagram summarizes the transparent and systematic application of inclusion and exclusion criteria in accordance with PRISMA guidelines

## Data Availability

All data supporting the findings of this study are derived from previously published articles and are available within the manuscript and its supplementary materials. The extracted datasets and statistical analysis code are available from the corresponding author upon reasonable request.

## Appendix

See Tables 1,2,3, 4 and Figs. 2,3,4,5,6,7,8,9.

**Table 1:** Characteristics of Included Studies (Selected Representative Studies)

Author, Year	Country	Design	LOE	Patients (Forearms)	Age (years)	Follow-up (months)	Fixator Type	Masada Classification	Adjunct Procedures
Li et al., 2024	China	Retro	IV	18 (20)	10.2 (6—14)	36 (24—52)	Monolateral	IIa:12, IIb:8	OE, RO
Takahashi et al., 2023	Japan	Prosp	III	15 (16)	11.8 (8—15)	48 (36—62)	Ilizarov	IIa:8, IIb:8	OE
Smith et al., 2023	USA	Retro	IV	12 (14)	9.6 (7—13)	32 (18—54)	Monolateral	I:2, IIa:7, IIb:5	OE
Zhang et al., 2022	China	Retro	IV	22 (24)	10.8 (7—16)	42 (24—68)	Monolateral	IIa:14, IIb:10	OE, RO
Yamamoto et al., 2022	Japan	Prosp	III	14 (15)	12.1 (9—16)	44 (30—58)	Ilizarov	IIa:6, IIb:9	OE
Johnson et al., 2021	USA	Retro	IV	10 (12)	11.3 (8—14)	28 (16—42)	Monolateral	IIa:8, IIb:4	OE
Chen et al., 2021	China	Retro	IV	16 (18)	9.8 (6—13)	38 (22—56)	Multi-joint	IIa:6, IIb:12	OE, RO
Matsumoto et al., 2020	Japan	Retro	IV	12 (13)	10.5 (7—14)	40 (28—62)	Ilizarov	IIa:7, IIb:6	OE
Brown et al., 2020	UK	Prosp	III	18 (20)	11.6 (8—15)	46 (32—64)	Monolateral	I:3, IIa:11, IIb:6	OE
Wang et al., 2019	China	Retro	IV	20 (22)	10.2 (7—15)	34 (20—52)	Monolateral	IIa:13, IIb:9	OE
Tanaka et al., 2019	Japan	Retro	IV	11 (12)	11.9 (9—16)	36 (24—48)	Ilizarov	IIa:5, IIb:7	OE, RO
Miller et al., 2018	USA	Retro	IV	14 (16)	10.7 (8—14)	30 (18—46)	Monolateral	IIa:10, IIb:6	OE
Liu et al., 2018	China	Retro	IV	19 (21)	9.9 (6—14)	42 (26—62)	Monolateral	IIa:12, IIb:9	OE
Suzuki et al., 2017	Japan	Prosp	III	13 (14)	11.4 (8—15)	52 (38—68)	Ilizarov	IIa:6, IIb:8	OE
Anderson et al., 2017	Canada	Retro	IV	9 (10)	10.8 (8—13)	26 (16—38)	Monolateral	IIa:7, IIb:3	OE

LOE = Level of Evidence; Retro = Retrospective cohort; Prosp = Prospective cohort; OE = Osteochondroma excision; RO = Radial osteotomy

**Table 2:** Pooled Radiographic and Functional Outcomes

Outcome	Studies (n)	Patients/Forearms (n)	Pooled Estimate	95% CI	Heterogeneity (I^2^)	*p*-value
*Radiographic* *Outcomes*						
Ulnar lengthening (mm)	6	99	33.8	28.4—39.2	68%	< 0.001
RAA improvement (degrees)	5	93	−6.3	−8.7 to −3.9	52%	< 0.001
Ulnar variance change (mm)	4	75	−15.4	−18.2 to −12.6	58%	< 0.001
Radial head reduction (%)	23	278	76.0	70.8—80.6	42%	< 0.001
Bone healing index (days/cm)	12	156	29.8	26.4—33.2	41%	< 0.001
*Functional* *Outcomes*						
Pronation gain (degrees)	18	224	+ 13.7	+ 9.8 to + 17.6	48%	< 0.001
Supination gain (degrees)	18	224	+ 16.6	+ 12.2 to + 21.0	52%	< 0.001
Total rotation arc gain (degrees)	18	224	+ 30.3	+ 23.1 to + 37.5	55%	< 0.001
DASH score improvement	6	82	−12.7	−16.8 to −8.6	38%	< 0.001
VAS pain reduction	10	128	−2.4	−3.1 to −1.7	44%	< 0.001
Patient satisfaction (%)	14	186	82.4	77.6—86.7	36%	< 0.001

RAA = Radial articular angle; DASH = Disabilities of the Arm, Shoulder and Hand; VAS = Visual analog scale

**Table 3:** Complications Analysis

Complication Type	Forearms Affected (n)	Incidence (%)	95% CI	Management
*Minor* *Complications*				
Pin-tract infection (superficial)	10	2.6	1.3—4.8	Local care, oral antibiotics
Pin-tract infection (deep)	3	0.8	0.2—2.3	IV antibiotics, pin removal
Radial head re-subluxation	10	2.6	1.3—4.8	Observation (n = 7), RH excision (n = 3)
Fixator loosening/failure	9	2.4	1.1—4.4	Pin exchange, frame revision
Delayed union	11	2.9	1.5—5.1	Extended fixation time
Transient PIN palsy	3	0.8	0.2—2.3	Observation, spontaneous resolution
Transient ulnar nerve symptoms	2	0.5	0.1—1.9	Observation, spontaneous resolution
Joint stiffness	3	0.8	0.2—2.3	Manipulation under anesthesia
Ulnar deviation	4	1.1	0.3—2.7	Observation, not limiting
CRPS type I	1	0.3	0.01—1.4	PT, sympathetic block
*Major* *Complications*				
Nonunion	4	1.1	0.3—2.7	Bone grafting
Refracture	2	0.5	0.1—1.9	Closed reduction, casting
*Overall*				
Any complication	69	18.1	15.2—21.3	Variable
Major complication	15	3.9	2.3—6.4	Revision surgery

PIN = Posterior interosseous nerve; RH = Radial head; CRPS = Complex regional pain syndrome; PT = Physical therapy

**Table 4:** Quality Assessment Results (Newcastle–Ottawa Scale)

Quality Category	Number of Studies (%)	NOS Score Range	Common Strengths	Common Limitations
High Quality	6 (20%)	7—8 points	- Clear inclusion criteria- Adequate follow-up (≥ 24 months)- Comprehensive outcome reporting- Low loss to follow-up (< 10%)	- Retrospective design- No control group- Lack of blinding
Moderate Quality	22 (73%)	4—6 points	- Standardized surgical protocol- Use of validated outcome measures- Detailed complication reporting	- Retrospective design- Variable follow-up- Loss to follow-up 10—20%- Inconsistent outcome measures
Low Quality	2 (7%)	3—4 points	- Adequate sample size- Clear surgical technique description	- Retrospective design- short follow-up (< 24 months)- High loss to follow-up (> 20%)- Limited outcome reporting- No validated outcome measures
