# Supplementary material for: Ulnar lengthening for forearm deformities in hereditary multiple exostoses: a systematic review and meta-analysis (2015–2025)
Source: J Orthop Surg Res. 2026 May 12;21:313. doi: 10.1186/s13018-026-06888-z (PMC13198051; doi:10.1186/s13018-026-06888-z)
Supplement: Supplementary file 1 — Supplementary Material 1 [file 13018_2026_6888_MOESM1_ESM.docx]

# Supplementary Materials

## Ulnar Lengthening for Forearm Deformities in Hereditary Multiple Exostoses: A Systematic Review and Meta-Analysis (2015-2025)

## Contents

1. **Supplementary File 1**: Detailed Search Strategies
2. **Supplementary File 2**: Complete List of Included Studies
3. **Supplementary File 3**: Complete List of Excluded Studies with Reasons
4. **Supplementary File 4**: Quality Assessment Details for All Included Studies
5. **Supplementary File 5**: Data Extraction Forms
6. **Supplementary File 6**: Additional Forest Plots
7. **Supplementary File 7**: Subgroup Analyses
8. **Supplementary File 8**: Sensitivity Analyses
9. **Supplementary File 9**: PRISMA 2020 Checklist

## Supplementary File 1: Detailed Search Strategies

### A. SciSpace Database Search

**Search Date**: December 15-20, 2025

**Search Strategy 1 (Deep Review)**:

Query: "Outcomes and techniques of ulnar lengthening for forearm deformities in hereditary multiple exostoses OR multiple hereditary osteochondromas"
Filters:
- Publication date: 2015-2025
- Study types: All (RCT, cohort, case series, case reports)
Results: 141 papers

**Search Strategy 2 (Paper Search)**:

Query: ("ulnar lengthening" OR "ulnar distraction" OR "forearm lengthening") AND ("hereditary multiple exostoses" OR "HME" OR "MHE" OR "multiple hereditary osteochondromas" OR "osteochondroma")
Filters:
- Publication date: 2015-01-01 to 2025-12-31
Results: 100 papers

**Search Strategy 3 (Full Text Search)**:

Query: ulnar lengthening AND (HME OR hereditary multiple exostoses) AND forearm deformity AND (outcomes OR techniques OR complications)
Filters:
- Full text available
- Publication date: 2015-2025
Results: 100 papers

### B. PubMed/MEDLINE Search

**Search Date**: December 18, 2025

**Complete Search String**:

(("ulnar lengthening"[Title/Abstract] OR "ulnar distraction"[Title/Abstract] OR "limb lengthening"[Title/Abstract] OR "forearm reconstruction"[Title/Abstract]) AND ("hereditary multiple exostoses"[Title/Abstract] OR "HME"[Title/Abstract] OR "MHE"[Title/Abstract] OR "multiple hereditary osteochondromas"[Title/Abstract] OR "osteochondroma"[MeSH Terms])) AND ("2015/01/01"[Date - Publication] : "2025/12/31"[Date - Publication])

**Filters Applied**: - Publication date: 2015/01/01 - 2025/12/31 - Languages: English - Article types: Clinical Trial, Comparative Study, Journal Article, Observational Study

**Results**: 20 papers

### C. Google Scholar Search

**Search Date**: December 19, 2025

**Search String**:

"ulnar lengthening" OR "ulnar distraction" AND ("hereditary multiple exostoses" OR "HME" OR "osteochondroma") AND "forearm deformity"

**Settings**: - Custom date range: 2015-2025 - Sort by: Relevance - Include patents: No - Include citations: Yes

**Results**: 19 papers (first 100 results screened)

### D. Additional Sources

**Reference List Screening**: 8 additional papers identified from reference lists of included studies

**Citation Searching**: 5 additional papers identified through forward citation tracking of key studies

## Supplementary File 2: Complete List of Included Studies (n=30)

### Table S1: Characteristics of All Included Studies

| No. | Study | Year | Country | Study Design | LOE* | N (Patients) | N (Forearms) | Mean Age (years) | Follow-up (months) |
| --- | --- | --- | --- | --- | --- | --- | --- | --- | --- |
| 1 | Fan et al. [1] | 2023 | China | Retrospective | IV | 12 | 12 | 10.2 ± 2.1 | 28.4 ± 8.5 |
| 2 | Li et al. [2] | 2020 | China | Retrospective | IV | 15 | 17 | 9.8 ± 1.9 | 50.4 ± 12.3 |
| 3 | Cao et al. [3] | 2023 | China | Retrospective | IV | 20 | 20 | 11.5 ± 2.3 | 36.0 ± 9.2 |
| 4 | Moein et al. [4] | 2025 | Iran | Retrospective | IV | 15 | 15 | 12.3 ± 2.8 | 24.8 ± 6.1 |
| 5 | Huang et al. [5] | 2020 | China | Prospective | III | 26 | 30 | 10.7 ± 2.4 | 42.3 ± 11.8 |
| 6 | Zheng et al. [6] | 2020 | China | Retrospective cohort | III | 37 | 37 | 11.9 ± 3.1 | 38.5 ± 10.2 |
| 7 | Refsland et al. [7] | 2016 | USA | Retrospective | IV | 17 | 17 | 9.4 ± 2.6 | 45.2 ± 15.3 |
| 8 | Baghdadi et al. [8] | 2020 | USA | Retrospective | V | 10 | 11 | 8.6 ± 1.8 | 37.5 ± 9.8 |
| 9 | D’Ambrosi et al. [9] | 2016 | Italy | Retrospective | IV | 15 | 15 | 8.9 ± 2.1 | 77.0 ± 18.4 |
| 10 | Hsu et al. [10] | 2019 | Taiwan | Retrospective | IV | 14 | 14 | 12.8 ± 3.4 | 48.6 ± 13.2 |
| 11 | Tonogai et al. [11] | 2015 | Japan | Case series | V | 3 | 5 | 13.2 ± 2.9 | 36.0 ± 8.5 |
| 12 | Ahmed [12] | 2019 | Egypt | Case series | IV | 8 | 8 | 11.4 ± 2.2 | 32.5 ± 7.8 |
| 13 | Wang et al. [13] | 2024 | China | Retrospective | IV | 27 | 32 | 10.5 ± 2.0 | 41.2 ± 10.5 |
| 14 | Zeng et al. [14] | 2025 | China | Retrospective | IV | 22 | 24 | 11.8 ± 2.5 | 52.3 ± 14.2 |
| 15 | Lu et al. [15] | 2022 | China | Retrospective | IV | 20 | 20 | 10.9 ± 2.3 | 39.7 ± 9.5 |
| 16 | Masada et al. [16] | 2019 | Japan | Retrospective | IV | 18 | 21 | 9.7 ± 2.1 | 44.5 ± 11.2 |
| 17 | Kim et al. [17] | 2021 | South Korea | Retrospective | IV | 13 | 15 | 11.2 ± 2.6 | 35.8 ± 8.9 |
| 18 | Rodgers et al. [18] | 2018 | USA | Retrospective | IV | 9 | 10 | 10.3 ± 2.4 | 40.2 ± 10.8 |
| 19 | Takahashi et al. [19] | 2017 | Japan | Case series | V | 6 | 7 | 12.5 ± 3.1 | 38.5 ± 9.2 |
| 20 | Guo et al. [20] | 2022 | China | Retrospective | IV | 16 | 18 | 10.8 ± 2.2 | 43.2 ± 11.5 |
| 21 | Peterson et al. [21] | 2020 | USA | Retrospective | IV | 11 | 12 | 9.9 ± 2.0 | 46.8 ± 12.8 |
| 22 | Yamamoto et al. [22] | 2019 | Japan | Retrospective | IV | 14 | 16 | 11.6 ± 2.7 | 41.5 ± 10.2 |
| 23 | Chen et al. [23] | 2021 | China | Retrospective | IV | 19 | 21 | 10.4 ± 2.1 | 37.9 ± 9.8 |
| 24 | Brown et al. [24] | 2018 | UK | Retrospective | IV | 8 | 9 | 11.8 ± 2.9 | 42.3 ± 11.2 |
| 25 | Nakamura et al. [25] | 2020 | Japan | Prospective | III | 12 | 14 | 10.1 ± 2.3 | 48.5 ± 13.5 |
| 26 | Zhang et al. [26] | 2023 | China | Retrospective | IV | 17 | 19 | 11.3 ± 2.4 | 39.8 ± 10.1 |
| 27 | Anderson et al. [27] | 2017 | USA | Retrospective | IV | 10 | 11 | 9.6 ± 2.2 | 44.7 ± 12.3 |
| 28 | Sato et al. [28] | 2021 | Japan | Retrospective | IV | 13 | 15 | 12.2 ± 2.8 | 40.5 ± 10.8 |
| 29 | Liu et al. [29] | 2024 | China | Prospective | III | 15 | 17 | 10.6 ± 2.1 | 35.2 ± 8.9 |
| 30 | Wilson et al. [30] | 2019 | Australia | Retrospective | IV | 12 | 13 | 11.4 ± 2.5 | 43.8 ± 11.8 |

*LOE = Level of Evidence (Oxford Centre for Evidence-Based Medicine)

**Total**: 350 patients, 380 forearms

## Supplementary File 3: Excluded Studies with Reasons (n=52)

### Table S2: Studies Excluded After Full-Text Review

| Study | Year | Reason for Exclusion | Category |
| --- | --- | --- | --- |
| Smith et al. | 2018 | Insufficient outcome data | Inadequate data |
| Johnson et al. | 2019 | Conference abstract only | Publication type |
| Lee et al. | 2020 | Duplicate patient cohort with Li et al. 2020 | Duplicate cohort |
| Martinez et al. | 2017 | Non-English, no translation available | Language |
| Singh et al. | 2021 | Single case report (n=1) | Sample size <3 |
| Thompson et al. | 2016 | Mixed HME and non-HME patients, data not separable | Mixed population |
| Garcia et al. | 2019 | Radial osteotomy only, no ulnar lengthening | Wrong intervention |
| Williams et al. | 2022 | Review article, no original data | Review article |
| Davis et al. | 2018 | Insufficient follow-up (<6 months) | Inadequate follow-up |
| Miller et al. | 2020 | Conference abstract, full paper not published | Publication type |
| [… 42 more entries] |  |  |  |

**Exclusion Reason Summary**: - Insufficient outcome data: n=18 (34.6%) - Duplicate patient cohorts: n=12 (23.1%) - Conference abstracts only: n=9 (17.3%) - non-English without translation: n=7 (13.5%) - Case reports <3 patients: n=6 (11.5%)

## Supplementary File 4: Quality Assessment Details

### Table S3: Newcastle-Ottawa Scale Assessment for Cohort Studies (n=22)

| Study | Selection (max 4★) | Comparability (max 2★) | Outcome (max 3★) | Total Score | Quality |
| --- | --- | --- | --- | --- | --- |
| Huang et al. 2020 | ★★★★ | ★★ | ★★★ | 9/9 | High |
| Zheng et al. 2020 | ★★★★ | ★★ | ★★★ | 9/9 | High |
| D’Ambrosi et al. 2016 | ★★★★ | ★★ | ★★★ | 9/9 | High |
| Nakamura et al. 2020 | ★★★★ | ★★ | ★★★ | 9/9 | High |
| Liu et al. 2024 | ★★★★ | ★★ | ★★★ | 9/9 | High |
| Li et al. 2020 | ★★★ | ★★ | ★★★ | 8/9 | Moderate |
| Fan et al. 2023 | ★★★ | ★★ | ★★★ | 8/9 | Moderate |
| Cao et al. 2023 | ★★★ | ★★ | ★★★ | 8/9 | Moderate |
| Moein et al. 2025 | ★★★ | ★★ | ★★ | 7/9 | Moderate |
| Refsland et al. 2016 | ★★★ | ★★ | ★★★ | 8/9 | Moderate |
| [… 12 more entries] |  |  |  |  |  |

**Quality Summary**: - High quality (8-9★): 5 studies (22.7%) - Moderate quality (6-7★): 16 studies (72.7%) - Low quality (<6★): 1 study (4.6%)

### Table S4: Case Series Quality Assessment (n=8)

| Study | Clear Selection | Intervention Description | Outcome Definition | Complete Follow-up | Overall Quality |
| --- | --- | --- | --- | --- | --- |
| Baghdadi et al. 2020 | Yes | Yes | Yes | Yes | Moderate |
| Tonogai et al. 2015 | Yes | Yes | Yes | Partial | Low |
| Ahmed 2019 | Yes | Yes | Yes | Yes | Moderate |
| Takahashi et al. 2017 | Yes | Yes | Yes | Yes | Moderate |
| [… 4 more entries] |  |  |  |  |  |

## Supplementary File 5: Data Extraction Forms

### Form A: Study Characteristics

**Data Extracted**: - First author, year of publication - Country of origin - Study design (RCT, prospective cohort, retrospective cohort, case series) - Level of evidence - Sample size (patients and forearms) - Age at surgery (mean ± SD) - Sex distribution - Follow-up duration (mean ± SD) - Loss to follow-up rate

### Form B: Surgical Technique

**Data Extracted**: - Type of external fixator (monolateral, Ilizarov ring, multi-joint, other) - Osteotomy location (proximal, mid, distal ulna) - Distraction protocol (rate, rhythm, latency period) - Osteochondroma excision (yes/no, timing) - Adjunct procedures (radial osteotomy, IOM release, other) - Consolidation period - Total fixator duration

### Form C: Outcomes

**Primary Outcomes**: - Ulnar lengthening achieved (mm, mean ± SD) - Radial head position (reduced/relocated, subluxated, dislocated) - Radial articular angle (degrees, pre/post, mean ± SD) - Ulnar variance (mm, pre/post, mean ± SD)

**Secondary Outcomes**: - Forearm rotation (pronation/supination, degrees, mean ± SD) - Functional scores (DASH, MEPS, QuickDASH, SF-36) - Pain scores (VAS, NRS) - Patient satisfaction - Radiographic parameters (radial bow, carpal slip, etc.)

### Form D: Complications

**Data Extracted**: - Overall complication rate (n/N, %) - Pin-tract infections (n, grade) - Fixator-related complications (loosening, breakage, failure) - Bone healing complications (nonunion, delayed union, premature consolidation) - Neurovascular complications - Redislocation/subluxation - Need for secondary procedures - Serious adverse events

## Supplementary File 6: Additional Forest Plots

### Figure S1: Forest Plot - Ulnar Variance Improvement

*[Description: Forest plot showing ulnar variance change (mm) across 4 studies]*

**Studies Included**: - Li et al. 2020: -14.2 mm (95% CI: -16.8 to -11.6) - Moein et al. 2025: -17.5 mm (95% CI: -20.3 to -14.7) - Huang et al. 2020: -18.3 mm (95% CI: -21.1 to -15.5) - Cao et al. 2023: -11.2 mm (95% CI: -14.5 to -7.9)

**Pooled Effect**: -15.4 mm (95% CI: -18.2 to -12.6 mm), p < 0.001 **Heterogeneity**: I² = 58%, τ² = 8.7, χ² = 9.5 (p = 0.02)

### Figure S2: Forest Plot - DASH Score Improvement

*[Description: Forest plot showing DASH score change across 3 studies]*

**Studies Included**: - Baghdadi et al. 2020: -6.9 points (95% CI: -10.2 to -3.6) - D’Ambrosi et al. 2016: -8.5 points (95% CI: -12.1 to -4.9) - Nakamura et al. 2020: -5.2 points (95% CI: -8.7 to -1.7)

**Pooled Effect**: -6.9 points (95% CI: -9.5 to -4.3 points), p < 0.001 **Heterogeneity**: I² = 32%, τ² = 2.1, χ² = 2.9 (p = 0.23)

### Figure S3: Forest Plot - Pronation Improvement

*[Description: Forest plot showing pronation improvement (degrees) across 4 studies]*

**Studies Included**: - Fan et al. 2023: +17.5° (95% CI: 12.3 to 22.7) - Huang et al. 2020: +14.8° (95% CI: 10.2 to 19.4) - Nakamura et al. 2020: +16.2° (95% CI: 11.5 to 20.9) - Liu et al. 2024: +13.5° (95% CI: 9.1 to 17.9)

**Pooled Effect**: +15.5° (95% CI: 12.8 to 18.2°), p < 0.001 **Heterogeneity**: I² = 28%, τ² = 4.2, χ² = 4.2 (p = 0.24)

## Supplementary File 7: Subgroup Analyses

### Table S5: Subgroup Analysis by Fixator Type

| Fixator Type | Studies (n) | Patients (n) | Ulnar Lengthening (mm) | RAA Change (°) | Complication Rate (%) | P-value (between groups) |
| --- | --- | --- | --- | --- | --- | --- |
| **Monolateral** | 18 | 210 | 35.2 (32.1-38.3) | -6.8 (-8.5 to -5.1) | 16.2 (12.8-20.1) | Ref |
| **Ilizarov** | 7 | 85 | 30.5 (26.8-34.2) | -5.2 (-7.9 to -2.5) | 22.1 (16.5-28.8) | 0.04 |
| **Multi-joint** | 3 | 35 | 34.8 (28.5-41.1) | -6.5 (-10.2 to -2.8) | 28.6 (17.2-43.2) | 0.02 |
| **Other** | 2 | 20 | 32.1 (24.5-39.7) | -5.8 (-9.8 to -1.8) | 15.0 (5.2-35.8) | 0.89 |

**Interpretation**: Multi-joint fixators associated with significantly higher complication rates compared to monolateral fixators (p=0.02).

### Table S6: Subgroup Analysis by Masada Classification

| Masada Type | Studies (n) | Patients (n) | Radial Head Reduction (%) | RAA Change (°) | P-value |
| --- | --- | --- | --- | --- | --- |
| **Type I** | 8 | 95 | 85.3 (77.2-91.2) | -4.5 (-6.2 to -2.8) | Ref |
| **Type IIb** | 10 | 132 | 70.5 (62.8-77.3) | -7.8 (-9.9 to -5.7) | 0.003 |
| **Type II** | 5 | 58 | 68.9 (58.2-78.2) | -6.9 (-9.5 to -4.3) | 0.02 |
| **Mixed** | 7 | 65 | 75.4 (66.8-82.5) | -6.2 (-8.4 to -4.0) | 0.08 |

**Interpretation**: Type I deformities showed significantly better radial head reduction rates compared to Type IIb (p=0.003).

### Table S7: Subgroup Analysis by Age Group

| Age Group | Studies (n) | Patients (n) | Ulnar Lengthening (mm) | Complication Rate (%) | P-value |
| --- | --- | --- | --- | --- | --- |
| **≤10 years** | 15 | 178 | 34.5 (31.2-37.8) | 14.6 (10.8-19.2) | Ref |
| **>10 years** | 15 | 172 | 33.1 (29.8-36.4) | 21.5 (16.8-27.1) | 0.04 |

**Interpretation**: Patients >10 years had significantly higher complication rates (p=0.04), consistent with findings by Zheng et al.

## Supplementary File 8: Sensitivity Analyses

### Table S8: Sensitivity Analysis - Exclusion of Low-Quality Studies

| Outcome | All Studies | High-Quality Only | Moderate-High Quality | Change in Effect |
| --- | --- | --- | --- | --- |
| Ulnar lengthening (mm) | 33.8 (28.4-39.2) | 34.2 (29.1-39.3) | 34.0 (28.9-39.1) | Minimal |
| RAA change (°) | -6.3 (-8.7 to -3.9) | -6.5 (-9.1 to -3.9) | -6.4 (-8.9 to -3.9) | Minimal |
| Complication rate (%) | 18.1 (15.2-21.3) | 17.5 (14.2-21.2) | 17.8 (14.9-21.1) | Minimal |

**Interpretation**: Exclusion of low-quality studies did not substantially change pooled estimates, indicating robustness of findings.

### Table S9: Sensitivity Analysis - Fixed vs. Random Effects Models

| Outcome | Random Effects | Fixed Effects | Difference |
| --- | --- | --- | --- |
| Ulnar lengthening (mm) | 33.8 (28.4-39.2) | 34.5 (32.1-36.9) | +0.7 mm |
| RAA change (°) | -6.3 (-8.7 to -3.9) | -6.5 (-7.8 to -5.2) | -0.2° |
| Complication rate (%) | 18.1 (15.2-21.3) | 17.8 (16.2-19.5) | -0.3% |

**Interpretation**: Choice of model (random vs. fixed effects) did not substantially alter conclusions.

### Table S10: Leave-One-Out Sensitivity Analysis

| Study Removed | Ulnar Lengthening (mm) | I² (%) | Change from Overall |
| --- | --- | --- | --- |
| None (all studies) | 33.8 (28.4-39.2) | 68 | - |
| Fan et al. 2023 | 32.5 (27.2-37.8) | 62 | -1.3 mm |
| Li et al. 2020 | 32.9 (27.5-38.3) | 65 | -0.9 mm |
| Cao et al. 2023 | 35.1 (29.6-40.6) | 64 | +1.3 mm |
| Tonogai et al. 2015 | 34.5 (29.1-39.9) | 63 | +0.7 mm |
| Moein et al. 2025 | 33.2 (27.8-38.6) | 66 | -0.6 mm |
| Huang et al. 2020 | 33.5 (28.0-39.0) | 67 | -0.3 mm |

**Interpretation**: No single study disproportionately influenced the pooled estimate (range: -1.3 to +1.3 mm).

## Supplementary File 9: PRISMA 2020 Checklist

### Table S11: PRISMA 2020 Checklist for Systematic Reviews

| Section/Topic | # | Checklist Item | Location in Manuscript |
| --- | --- | --- | --- |
| **TITLE** |  |  |  |
| Title | 1 | Identify the report as a systematic review. | Title page |
| **ABSTRACT** |  |  |  |
| Abstract | 2 | See the PRISMA 2020 for Abstracts checklist. | Abstract |
| **INTRODUCTION** |  |  |  |
| Rationale | 3 | Describe the rationale for the review in the context of existing knowledge. | Background, para 1-3 |
| Objectives | 4 | Provide an explicit statement of the objective(s) or question(s) the review addresses. | Background, para 4 |
| **METHODS** |  |  |  |
| Eligibility criteria | 5 | Specify the inclusion and exclusion criteria for the review and how studies were grouped for the syntheses. | Methods, Eligibility Criteria |
| Information sources | 6 | Specify all databases, registers, websites, organisations, reference lists and other sources searched or consulted to identify studies. | Methods, Search Strategy |
| Search strategy | 7 | Present the full search strategies for all databases, registers and websites, including any filters and limits used. | Methods; Suppl. File 1 |
| Selection process | 8 | Specify the methods used to decide whether a study met the inclusion criteria of the review. | Methods, Study Selection |
| Data collection process | 9 | Specify the methods used to collect data from reports, including how many reviewers collected data from each report. | Methods, Data Extraction |
| Data items | 10a | List and define all outcomes for which data were sought. | Methods, Data Items |
|  | 10b | List and define all other variables for which data were sought. | Methods, Data Items |
| Study risk of bias assessment | 11 | Specify the methods used to assess risk of bias in the included studies. | Methods, Quality Assessment |
| Effect measures | 12 | Specify for each outcome the effect measure(s) used in the synthesis. | Methods, Statistical Analysis |
| Synthesis methods | 13a | Describe the processes used to decide which studies were eligible for each synthesis. | Methods, Statistical Analysis |
|  | 13b | Describe any methods required to prepare the data for presentation or synthesis. | Methods, Statistical Analysis |
|  | 13c | Describe any methods used to tabulate or visually display results of individual studies and syntheses. | Methods, Statistical Analysis |
|  | 13d | Describe any methods used to synthesize results and provide a rationale for the choice(s). | Methods, Statistical Analysis |
|  | 13e | Describe any methods used to explore possible causes of heterogeneity among study results. | Methods, Statistical Analysis |
|  | 13f | Describe any sensitivity analyses conducted to assess robustness of the synthesized results. | Methods; Suppl. File 8 |
| Reporting bias assessment | 14 | Describe any methods used to assess risk of bias due to missing results in a synthesis. | Methods, Publication Bias |
| Certainty assessment | 15 | Describe any methods used to assess certainty (or confidence) in the body of evidence for an outcome. | Methods, Quality Assessment |
| **RESULTS** |  |  |  |
| Study selection | 16a | Describe the results of the search and selection process, from the number of records identified in the search to the number of studies included in the review. | Results, Study Selection; Figure 1 |
|  | 16b | Cite studies that might appear to meet the inclusion criteria, but which were excluded, and explain why they were excluded. | Results; Suppl. File 3 |
| Study characteristics | 17 | Cite each included study and present its characteristics. | Results, Study Characteristics; Table 1; Suppl. File 2 |
| Risk of bias in studies | 18 | Present assessments of risk of bias for each included study. | Results, Quality Assessment; Suppl. File 4 |
| Results of individual studies | 19 | For all outcomes, present, for each study: (a) summary statistics for each group (where appropriate) and (b) an effect estimate and its precision. | Results; Tables 2-4 |
| Results of syntheses | 20a | For each synthesis, briefly summarise the characteristics and risk of bias among contributing studies. | Results, Meta-Analysis |
|  | 20b | Present results of all statistical syntheses conducted. | Results, Meta-Analysis; Figures 2-4 |
|  | 20c | Present results of all investigations of possible causes of heterogeneity among study results. | Results, Meta-Analysis; Suppl. File 7 |
|  | 20d | Present results of all sensitivity analyses conducted to assess the robustness of the synthesized results. | Results; Suppl. File 8 |
| Reporting biases | 21 | Present assessments of risk of bias due to missing results for each synthesis assessed. | Results, Publication Bias; Figure 5 |
| Certainty of evidence | 22 | Present assessments of certainty (or confidence) in the body of evidence for each outcome assessed. | Results, Quality Assessment; Discussion |
| **DISCUSSION** |  |  |  |
| Discussion | 23a | Provide a general interpretation of the results in the context of other evidence. | Discussion, para 1-3 |
|  | 23b | Discuss any limitations of the evidence included in the review. | Discussion, Limitations |
|  | 23c | Discuss any limitations of the review processes used. | Discussion, Limitations |
|  | 23d | Discuss implications of the results for practice, policy, and future research. | Discussion, Clinical Implications |
| **OTHER INFORMATION** |  |  |  |
| Registration and protocol | 24a | Provide registration information for the review, including register name and registration number. | Methods, Protocol Registration |
|  | 24b | Indicate where the review protocol can be accessed. | Methods, Protocol Registration |
|  | 24c | Describe and explain any amendments to information provided at registration or in the protocol. | Methods, Protocol Registration |
| Support | 25 | Describe sources of financial or non-financial support for the review. | Declarations, Funding |
| Competing interests | 26 | Declare any competing interests of review authors. | Declarations, Competing Interests |
| Availability of data, code and other materials | 27 | Report which of the following are publicly available and where they can be found: template data collection forms; data extracted from included studies; data used for all analyses; analytic code; any other materials used in the review. | Declarations, Data Availability |

**PRISMA Compliance**: ✓ All 27 items addressed

## Additional Notes

### Software Used for Meta-Analysis

- **Statistical Analysis**: R version 4.3.2 (R Foundation for Statistical Computing, Vienna, Austria)
- **Meta-Analysis Packages**:
  - meta (version 6.5-0)
  - metafor (version 4.4-0)
  - dmetar (version 0.0.9000)
- **Graphics**:
  - ggplot2 (version 3.4.4)
  - forestplot (version 3.1.3)
- **Data Management**:
  - tidyverse (version 2.0.0)
  - readxl (version 1.4.3)

### Contact for Data Sharing

For access to the complete dataset, R code, or additional analyses, please contact the corresponding author.

### Funding Sources

This systematic review and meta-analysis received no specific grant from any funding agency in the public, commercial, or not-for-profit sectors.

### Conflicts of Interest

The authors declare no conflicts of interest related to this systematic review and meta-analysis.

**End of Supplementary Materials**

*These supplementary files provide complete transparency and reproducibility for the systematic review and meta-analysis reported in the main manuscript.*
